# Just like the rest of evolution in Mother Nature, the evolution of cancers may be driven by natural selection, and not by haphazard mutations

**DOI:** 10.18632/oncoscience.83

**Published:** 2014-09-22

**Authors:** Ju Zhang, Xiaomin Lou, Lucas Zellmer, Siqi Liu, Ningzhi Xu, D. Joshua Liao

**Affiliations:** ^1^ CAS Key Laboratory of Genome Sciences and Information, Beijing Institute of Genomics, Chinese Academy of Sciences, Beijing 100101, P.R. China; ^2^ Hormel Institute, University of Minnesota, Austin, MN 55912, USA; ^3^ Laboratory of Cell and Molecular Biology, Cancer Institute, Chinese Academy of Medical Science, Beijing 100021, P.R. China

**Keywords:** evolution, cancer, oncogenes, tumor suppressor genes, mutation

## Abstract

Sporadic carcinogenesis starts from immortalization of a differentiated somatic cell or an organ-specific stem cell. The immortalized cell incepts a new or quasinew organism that lives like a parasite in the patient and usually proceeds to progressive simplification, constantly engendering intermediate organisms that are simpler than normal cells. Like organismal evolution in Mother Nature, this cellular simplification is a process of Darwinian selection of those mutations with growth- or survival-advantages, from numerous ones that occur randomly and stochastically. Therefore, functional gain of growth- or survival-sustaining oncogenes and functional loss of differentiation-sustaining tumor suppressor genes, which are hallmarks of cancer cells and contribute to phenotypes of greater malignancy, are not drivers of carcinogenesis but are results from natural selection of advantageous mutations. Besides this mutation-load dependent survival mechanism that is evolutionarily low and of an asexual nature, cancer cells may also use cell fusion for survival, which is an evolutionarily-higher mechanism and is of a sexual nature. Assigning oncogenes or tumor suppressor genes or their mutants as drivers to induce cancer in animals may somewhat coerce them to create man-made oncogenic pathways that may not really be a course of sporadic cancer formations in the human.

Carcinogenesis starts from the reprogramming of a somatic cell’s death program, making the cell immortalized and autonomous. Sporadic carcinogenesis, a process for cancer formation in adults, may originate from a cessation of differentiation of an organ-specific stem cell [1,2] or from de-differentiation of an already differentiated cell. Childhood carcinogenesis has likely begun during the embryonic stage when cells have not yet differentiated or not yet fully differentiated, and thus more likely involves a stopping of differentiation of an embryonic cell. Forming a “tumor”, the progenies of the immortalized cell may or may not proceed to simplification, a reverse evolution [3] that is also dubbed as atavism in the literature [4]. If such cellular simplification does not occur, the tumor, which could be benign, will likely not progress; otherwise it will continuously result in phenotypes of more aggressiveness, including invasion, metastasis and therapy-resistance. In such a sporadic cancer that originates from a differentiated cell, cellular simplification must be involved, as cancer is less differentiated than normal cells in both morphology and function [3,4]. The immortalized cell actually commences a new or quasi-new organism, as originally described by David Hansemann in 1897 in his book *Die mikroskopische Diagnose bösartiger Geschwülste* [5] and later emphasized by renowned evolutionist Huxley who wrote in 1956 that “all autonomous neoplasms can be regarded as the equivalents of new biological species” [6]. This is because the tumor as an entity is not only immortal but also autonomous, i.e. no longer loyal to the host animal as expounded in more detail recently [7], whereas all normal cells in the patient give their allegiance to the body and will eventually die. This “new organism” lives in the patient like a parasite, as put by Vincent [4], and the continuously emerging lesions of more aggressiveness, resulting from its cellular simplification, resemble individual new organisms that are simpler than normal cells [4,7].

Immortalization of organ- or tissue-specific stem cells that leads to a stop in differentiation, and immortalization of already-differentiated cells that later leads to de-differentiation, may all involve genetic alterations [8], which are herein collectively referred to as “DNA mutations” for simplicity. In some cases, especially of pediatric cancer, the mutation may be inherited, i.e. it exists in one or both parental germ cells. The ensuing simplification also involves mutations. Pertaining to how these mutations contribute to progressive carcinogenesis, we favor Blagosklonny’s opinion [9] but have different meditations from the mainstreams of cancer research. In this essay, we present our musings on this and several other issues while avoiding details in some basic profiles that are already familiar to most peers. “DNA mutation”, but not “gene mutation”, is used here because the human exome, the part of the genome that encodes amino acids in proteins, only constitutes slightly over 1% of the human genome. However, virtually all the non-repeat part of the genome is transcribed [10-14], thus leaving the remaining near 99% being non-coding but probably still relevant to carcinogenesis.

## Organ-specific stem cells protect the organ from developing cancer

**Figure 1:**
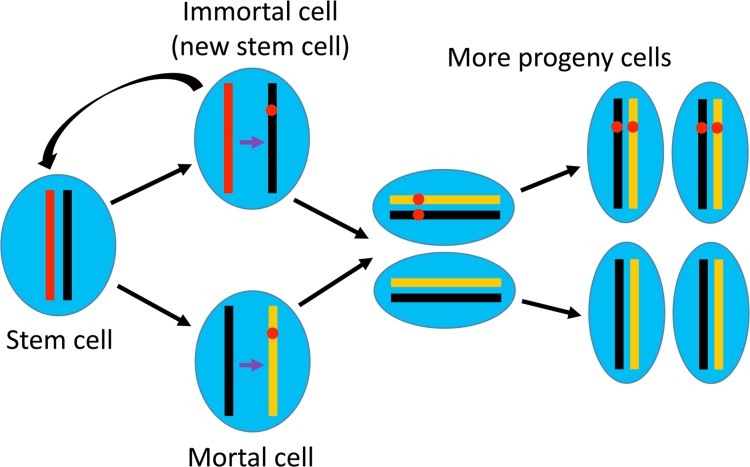
Hypothetical asymmetric segregation of the mortal and immortal DNA strands in organ-specific stem cells Of the two strands of DNA double helix in any cell, one is old, inherited (conserved) from the parental cell (red bar) while the other is new (black bar), because DNA synthesis is semiconservative. During division of an organ-specific stem cell, one daughter cell is immortal as it becomes a stem cell again, like its parental cell, whereas the other is mortal as it will continue proliferation to generate more progeny cells. After semiconservative synthesis (indicated by a purple arrow), the new DNA double helix (red and black bars) that contains the old template strand is always passed to the new stem cell, whereas the other new double helix (black and yellow bars) that contains the new template strand is always passed to the mortal cell that will undergo routine cell division as other somatic cells. Therefore, if a mutation occurs (red dot in the yellow bar that represents the newer DNA strand) in the mortal daughter cell or its progenies, it can be double-stranded and thus fixed in DNA in newer progeny cells. In contrast, if a mutation occurs in the immortal daughter cell (red dot in the black bar), it will not be fixed for a long time because the cell only divides occasionally, and, once it divides again, the mutation will only be passed to its mortal daughter cell.

Short-lived animals are evolutionarily lower in the life tree and lack tissue- or organ-specific stem cells or even highly specialized tissues or organs, albeit they may still develop tumors or even cancers [15]. Therefore we wonder why long-lived animals have evolved stem cells in those organs that have a routine cell turnover, especially those having external or luminal surfaces such as the skin, prostate, breasts, lungs, as well as the gastrointestinal tract (e.g. esophagus, stomach and colon) and glands (the liver and pancreas), where most human cancers arise. As expounded by Cairns [16,17], the evolutionary development of organ-specific stem cells protects, by continuous cell replacement, the organs from carcinogenesis induced by various physical (e.g. radiation), chemical (e.g. carcinogens) or biological (e.g. viruses) factors. These stem cells occasionally undergo asymmetric division, engendering one immortal daughter cell that is identical to the parental one and stays in a quiescent status for most of the time and one mortal daughter cell that continues replicating to meet the routine cell turnover demand. During this asymmetric division, the immortal daughter cell always receives the old strand of the DNA double helix whereas the mortal daughter cell always receives the new DNA strand (Figure 1), which prevents mutations from being double-stranded and then fixed in stem cells [18,19]. This asymmetric division with asymmetric segregation of sister chromatids purges mutations from the organ and thus prevents cancer formation, because the cell turnover removes most cells before they have accumulated enough mutations to be immortalized and beyond. This is one reason, besides many others such as immune surveillance [20,21], why sporadic cancer formation usually requires about one-fourth or more of the life span, which in humans translates to about 20 years.

Whether division of cancer stem cells also has an asymmetric segregation of sister chromatids with the immortal DNA strand always in the immortal daughter cell is an intriguing question hardly being addressed hitherto, to our knowledge. There has never been a lucid definition of “cancer stem cells” in the literature to clearly distinguish it from normal stem cells and from the vast majority of cancer cells. If such asymmetric segregation of the two DNA strands is retained in some cancer cells, it may be a strong evidence for the existence of cancer stem cells; otherwise “cancer stem cells” is just an *ad hoc* concept to describe those cancer cells that have a stronger growth- or survival-potential or a greater differentiation potential [22]. All pathologists who read cancer slides know that cancers have a much higher cell death toll than their adjacent normal tissues, even when central necrotic areas are excluded. Of those still-alive cancer cells, some have much weaker health and thus have weaker growth or survival ability than the others, which, however, does not entitle those with a greater ability to grow, such as in soft agar and immunodeficient mice, to be “cancer stem cells” as described in many reported studies [23].

About 12% of the single-nucleotide polymorphisms in the human genome are actually harmful [24], making it possible that some mutations may actually be more beneficial than the wild type [24-26]. This conjecture leads to an intriguing question as to whether cells can convert some mutations back to the wild type, especially when the microenvironment that favors cancer formation returns to normal, such as when environmental pollution has been controlled or when medical management is applied. This is a reasonable question because such “back mutation” or “reverse mutation” of inherited mutations has been well documented for some non-cancerous diseases [27-30]. Moreover, secondary mutations to override the adverse effects of a prior mutation have been observed for the BRCA1 and BRCA2 genes after chemotherapy of some cancers [31-33]. Therefore, there may be a short window during carcinogenesis for such reverse or secondary mutations to occur, either spontaneously or by manipulation, leading to abortion of the carcinogenesis.

## Gain of oncogenes and loss of tumor suppressor genes may be results of Darwinian selections

There are two major but quite different hypotheses on how genetic alterations cause cancer. One hypothesis, which has received much less recognition, considers that carcinogenesis involves mutations that emerge in a random and stochastic manner, making cancer formation completely unpredictable [34-36]. Another hypothesis is that carcinogenesis is a course of sequential gene mutations that confer onto the cell growth advantage and thus are called “drivers”, although different cell types have different successions of driver mutations [37-39]. The latter hypothesis, which has been the dominant and been firmly entrenched in the cancer research society for decades, often uses colorectal carcinogenesis as a paradigm wherein the *APC* gene mutation is identified as a driver [38,39]. Surprisingly, sequencing thousands of cancer samples leads to identification of only an average of 33-66 genes in a common solid tumor, or a total of 138 genes (74 tumor suppressor genes and 64 oncogenes) for many different malignancies, that have driver mutations. In contrast, 18,306 genes (roughly 90% of the about 20,000 genes in the genome [10,11]) are found to have a total of 404,863 mutations [37,40], with one cancer having 1,000-5,000 mutations [41-43]. Besides those in the 138 genes, the vast majority of mutations in other genes are considered “passengers”, i.e. are those irrelevant to carcinogenesis [37,42,43]. This situation is in line with Prehn’s earlier description of “cancers beget mutations versus mutations beget cancer” [44]. It should be pointed out that there are other hypotheses, such as the so-called ‘tissue organization field theory (TOFT)” that does not consider mutations in somatic cells as the drivers of cancer but, instead, believes that cancer is a problem of tissue organization irrelevant to mutations [45-48]. This TOFT theory has been disputed in the literature [49] and thus will not be discussed herein.

In our cogitation, the number of genes that can serve as the drivers of carcinogenesis is huge, far more than the abovementioned 138 genes with driver mutations [37], if the definition of “driver” is extended to include a gene’s wild type form that has an ability to confer a growth or survival advantage. We hypothesize that many driver genes may not need to be mutated during the initial stage of carcinogenesis, because their aberrant expression is sufficient to drive cell replication. However, mutation will occur later as happenchance in more and more genes at a quicker and quicker speed while the carcinogenesis proceeds. The numerous mutations that emerge in a random and stochastic manner, as described by Heng et al [34-36], spread widely in numerous cells. As the result, each individual cell has only a few mutations, and even fewer if only those are counted that render to the gene (usually tumor suppressor genes like p53) an ability to promote cell growth or survival. Moreover, many mutations no longer exist at the time of detection, because the mutations have been repaired or the mutation-bearing cells have already died. Some cellular deaths occur because some mutations are incompatible to the others.

**Figure 2:**
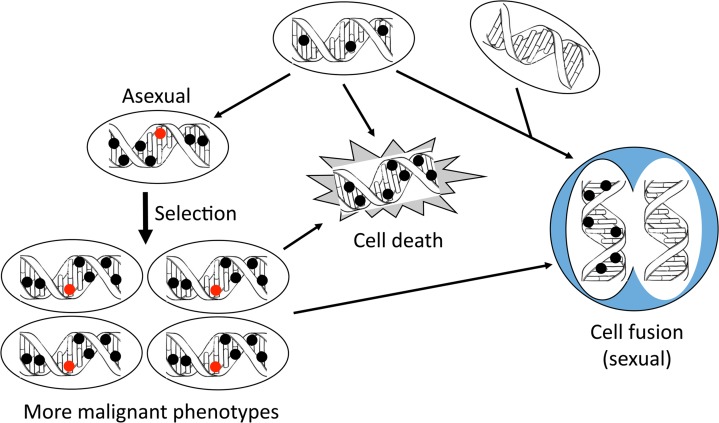
Asexual and sexual manners of cancer evolution Mutations (dark and red dots) occur randomly and stochastically in cancer cells. On one hand, accumulation of mutations will lead to the loss of too many life-sustaining genes, in turn causing some cell deaths. On the other hand, some beneficial mutations (red dot), i.e. those with survival- or growth-advantages, will emerge and then be selected via clonal expansion to manifest a phenotype that usually is more aggressive than their progenitor cells. This mutation-load dependent mechanism for survival is asexual and usually used by evolutionarily-lower organisms such as bacteria. Some of the clonally expanded cells may still die later, due to losing more genes. Along with the cell deaths and asexual propagations is yet another way of survival, in which some cancer cells choose to fuse with another cell, such as a normal stromal cell that has no mutation. This cell fusion resembles fertilization of an egg by a sperm and thus is similar to a sexual propagation usually used by evolutionarily-higher organisms to gain beneficial mutations while purging away deleterious mutations. Indeed, the resulting hybrid cell receives beneficial genetic material and usually is more malignant, such as being more potent in metastasizing. Actually, some of the clonally expanded cells may also later take this way of survival by fusing with another cell.

Natural selection is actually a course of clonal expansion of those cells that have functional gain of oncogenes and/or functional loss of tumor suppressor genes, either of the two rendering the cells growth- or survival-advantage in their particular microenvironments (Figure 2). Therefore, a best known molecular profile of cancer cells, i.e. functional gain of survival- or growth-sustaining oncogenes and functional loss of differentiation-sustaining tumor suppressor genes, is actually a result of natural selection from numerous mutations that emerge randomly and stochastically. Restated more clearly, these common alterations of cancer are not the drivers of cancer formation or progression as regarded by the mainstream of cancer research [37,50] but, instead, are the results of natural selections from numerous mutations occurring as happenstances. However, selection has to wait for its chance, i.e. wait until an advantageous mutation or even a set of synergistic mutations have emerged, and God knows how long it will take. Therefore, functional gain of an oncogene or functional loss of a tumor suppressor gene, and the ensuing selection via clonal expansion, take a long time and occur as an evolution, which is another reason why sporadic cancer formation takes many years.

Our hypothesis, which considers that the common traits of cancer result from natural selections from randomly and stochastically occurring mutations, to some extent merges together the two existing hypotheses described above. Moreover, our hypothesis seems to be a compromise with the abovementioned TOFT theory as well [45-48], because, like TOFT, it does not consider mutations as the drivers but, instead, as the results of carcinogenesis. However, our hypothesis acknowledges the importance of mutations in cancer cells’ behaviors and as possible prognostic markers and therapeutic targets [51], since the mutations are elected to dictate most cells’ biology. Actually, the TOFT theory does not really reject the roles of mutations in carcinogenesis in many aspects, as having been clearly pointed out by Blagosklonny [49]. Natural selection of random mutations may also explain heterogeneity in the same type of cancer among different patients. For instance, tumors in different patients may select different genes along the ERK/MAPK pathway for a growth- or survival-advantage; as a result, these tumors show heterogeneity in gene mutation but all show the activation of the ERK/MAPR survival pathway.

## Aggressive phenotypes also result from natural selection of random mutations

Carcinogenesis as a continuous process is well known to result in cells of greater and greater malignancy, manifested as invasion to the surrounding tissue, metastasis to distant body sites, and resistance to therapies. These occur presumably because individual cells accumulate more DNA mutations, which in turn is because the cells progressively lose their DNA damage response and their DNA repair mechanisms, and thus have ever greater difficulty in repairing continually emerging mutations. Natural selection ensues to select, from all these mutations, those with growth- or survival-advantages, usually occurring in oncogenes or tumor suppressor genes (Figure 2), as aforementioned. This type of selection somewhat resembles organismal development during Darwinian selection in Mother Nature. However, Mother Nature changes only very slowly, over millions of years, and thus organismal evolution also occurs slowly. In contrast, the larger and larger tumor mass and the reciprocally weaker and weaker patient’s health together change the cancer’s microenvironment more and more rapidly, and thus cell clones with greater and greater diversity are selected more and more quickly. Actually, the tissue environment can change in just minutes or hours, especially in the presence of therapies, requiring cancer cells to change accordingly, as it is a matter of life or death for them. For instance, the friendly environment around cancer cells is immediately shifted to a very hostile one during radiation therapy, in less than the second needed to switch on the radiation device. The cancer cells have to immediately mobilize their wherewithal to adapt to the vicious environment, likely with quicker, non-genetic approaches first, such as protein phosphorylation or RNA editing. It can be imagined that some cancer cells may choose to phosphorylate an oncoprotein while other cells cannot do so, because the gene coding for the kinase or the oncoprotein is mutated or silenced, and thus choose to edit another gene’s mRNA instead [52]. These disparities are collectively manifested as a heterogeneity of survived cells and selected clones. Moreover, this somatic cell evolution occurs in an asexual manner, as it increases the cellular heterogeneity by mutation loads, in contrast to the sexual propagation in which sex functions to purge altered genome to maintain the species identity, as explained later (Figure 2). Because of this fundamental difference, asexual propagation of cells widens heterogeneity of the progenies, whereas sexual propagation maintains the species no matter how many generations have passed [34,35].

As explained elsewhere [7], progressive simplification of cancer cells continuously yields simpler cells that resemble “intermediate organisms”, although this process is considered by others as an ongoing speciation event of a unicellular eukaryotic species [5,53]. Cells of these “intermediate organisms” initially still retain some simple tissue structure such as ducts or glands (ductal or glandular carcinomas), but later completely lose tissue organization and become individual invasive cells disseminating in the surrounding stromal tissue, somewhat similar to unicellular protozoans [7]. Distant metastasis ensues, probably in part because the microenvironment of the new body site is less stressful or because the microenvironment of the original tissue or organ is no longer suitable for these even simpler “organisms” [4]. Metastases usually appear as multiple masses, each of which may be considered a new organism that lives like a parasite. For this reason, a cancer patient is “parasitized” by not just a single type, but by multiple types, of “parasites”. Moreover, some cancer cells continue losing their genes by mutations or by epigenetic inactivation such as methylation, because the cells no longer need specialized functions, such as liver cancer’s loss of bile production. What are also lost are some self-protective mechanisms. These features of simpler organisms, which could be used as therapy targets, make cancer cells more fragile to various forms of stress, compared with their normal counterparts. Also similar to simpler organisms, cancer cells may have a shorter life span relative to the normal counterparts in the host tissue or organ, which is one of the reasons why cancers manifest a much higher cell death toll than the corresponding normal tissue [7,54]. Actually, cell death is sometimes a criterion of malignancy, such as for breast cancer and some sarcomas [55]. In contrast, long life-span cells such as striated muscles relatively rarely develop sporadic cancer, and heart muscles even much more rarely do so.

The recent results from high throughput sequencing of thousands of cancer samples show that basically all mutations found in advanced tumors can also be found in the primary tumors [37]. These results, although they have already been predicated by Blagosklonny over a decade ago [9], seem to challenge the above “Darwinian selection” hypothesis for how more-aggressive phenotypes occur. Our explanations for this seeming incongruity are twofold. First, some phenotypes of greater aggressiveness, such as resistance to therapies, may appear initially via altered gene expression, but not through mutations [56,57]. As aforementioned, the microenvironment may be changed in just a second, and thus cancer cells accordingly have to equip themselves with some quick response mechanisms. Some of these mechanisms may be maintained for a long time, and may or may not be succeeded by a relatively stable epigenetic mechanism but will eventually be superseded by mutation as a permanent mechanism. Second, although a primary tumor mass has been detected for all mutations needed for a phenotype of more malignancy, it does not mean that there has been a cell that bears all these mutations. The tumor still needs time to allow some individual cells to accumulate all the mutations required for the presentation of a moreaggressive phenotype. Equipping a single cell with all the required mutations is just the phase I of the evolution, which will be followed by a phase II that is the natural selection via clonal expansion of this cell to present its particular phenotype, such as chemo-resistance. Several cancers are highly curable, such as testicular cancer and gestational choriocarcinoma, likely because they arise without lengthy selection and progression, as inferred by Blagosklonny [58]. Metastasis-favoring mutations certainly occur first in some cells of the primary tumor to allow the cells later to break away from the tumor mass and lodge in another body site. In addition, the differences in mutations between primary and metastatic or between therapy-sensitive and -resistant tumors are probably quantitative rather than qualitative. A mutation is detected because it appears in more cells, which is in turn because of the clonal expansion during natural selection.

## Putting the cart before the horse leads to many “oncogenic pathways” made or to be made

For 80 years it has been known that sex steroids can induce histologically confirmed cancer in multiple organs of animals, but, at least at some stages, most of the tumors completely regress after withdrawal of the hormones [59-66]. Some cancers induced by transgenes, such as in some strains of c-*myc* and/or k-*ras* transgenic mice [67-69], also regress after inactivation of the transgenes. This phenomenon is more often referred to as “oncogene addition” or “tumor dormancy” in recent literature ([70-72] and reference in [73]), because re-administration of the inducer can quickly induce the tumor again. Actually, induced senescence has also been shown to be reversible upon withdrawal of the inducer [72,74-76], although spontaneous senescence should be irreversible. The inducer-dependent cancers are different from carcinogen-induced ones that, once becoming malignant, no longer require the presence of the carcinogens. Therefore, the ultimate drivers in these inducer-dependent models are not any of those genes that are mutated in the tumors, but are the exogenous chemicals (e.g. sex steroids) or the ectopic transgenes. One may argue that these inducer-dependent lesions, at least the vast majority of their cells, are not really cancerous, not even benign by definition in tumor biology, not only because they differ from human cancers that rarely completely regress spontaneously but also because these cells are not immortal, albeit they are morphologically malignant. Indeed, at least in some cases the tumor cells are still mortal when cultured in a dish [77] and thus are not even qualified to be benign, as already explained before [73].

The only reliable criterion for distinguishing cancers from benign tumors is their ability to metastasize [78], although in reality pathologists have to diagnose immediately whether a surgically-removed tumor is malignant or benign based on its histology and cannot hold on the diagnosis until the patient later shows or does not show metastasis. This actually says that most of those well-known “cancer hallmarks” proposed by Hanahan and Weinberg [50,79] are only tumor hallmarks, but not cancer hallmarks, as having been clearly pointed out by Lazebnik [78]. Or, in Blagosklonny’s words, “hallmarks can be observed without cancer” [80]. Tumors developed in a majority of genetically engineered animal models cannot metastasize, but yet the tumors are still claimed to be “cancer” [78], which actually announces that “a large percentage, if not the majority, of solid cancers can be cured simply by surgical removal, no matter how advanced they are.” Fortunately (or unfortunately), we still have jobs today because this is only true in these animals, but not in the humans. In our opinion, this key disparity greatly diminishes the human relevance of many animal models, no matter how histologically similar the resulting tumors are to the human counterparts, since it is the metastases that usually are multiple, inoperable lesions and eventually kill the patient. In a nutshell, the three common features, i.e. the mortality in Petri dish, the inducer-dependency and the inability to metastasize, question the cancerous identity of the tumors from many animal models, and have created huge hurdles on the bridge leading cancer molecular biology to cancer biology, although these models have indeed helped in delineating many molecular details of cancer.

Nice persons may also commit outrageous crimes if they are coerced by truly bad guys, but, once they are free of the coercion, they will likely be model citizens again as they once were. In those inducer-dependent animal models, the inducers (steroids, transgenes, etc.) may just act as coercions, and the cancer morphology does not really reflect the whole truth as it is only being sustained by coercion. Probably, when some genes or their mutants are assigned by us as the drivers to induce cancers in animals, they may actually be coerced to be the “criminals” and thus are wronged. In most canonical transgenic animal models, the transgene is constantly active, thus constantly sustaining the cancer morphology. In some models, the DNA promoter has already been active during an early embryonic stage, such as the Mist1 promoter [81], driving the transgene to maintain the cancer morphology as poorly differentiated as are early embryonic cells [82]. As having been pointed out earlier [82], the time point at which the DNA promoter starts to activate the transgene matters but has rarely been discussed in the literature on transgenic models.

As aforementioned, cancers are characterized by their gained function of oncogenes and lost function of tumor suppressor genes. Misplacing these alterations as the drivers of carcinogenesis, peers have identified some, but have created many more, pathways leading from a normal to a cancerous state by engineering genes in animals. For instance, one can establish an animal model by simultaneously expressing a c-*myc* and a *ras* oncogene, a second model by expressing the c-*myc* first and then the *ras*, and a third model by expressing the *ras* first and then the c-*myc* [73]. There are at least 100 genes critical to cancer, and hitting two of them may lead to carcinogenesis, according to the so-called “two hits” principle [83-85]. Therefore, theoretically there should be 3×2×100(100-1)/2 (=29700) animal models or pathways of carcinogenesis that can be made by us, if each gene is engineered in two opposite (transgenic and knockout) ways. The figure can even be much huger since in many models three or more genes are manipulated, although Vogelstein et al summarize only 12 pathways [37], in part because many genes are linear in the same pathways. Indeed, a huge number of animal models have been made using genetic-engineering technology. As an analogy, we can build, as many as we would like, one-way pathways leading from Chicago (considered “normal”) to Detroit (considered “cancer”), as long as we don’t have to be concerned that anyone would actually like to travel on any of them. For instance, a *Tert* transgene is often utilized to immortalize cells in animals [73]. The results are used to hint delicately, but never to pronounce, that “altered *Tert* is a driver of human cancer”. Peers can survive in today’s funding gloom by taking this “me too” approach, i.e. by continuing to engineer mice, two or three genes per animal, to create new strains of mice showing new oncogenic pathways. Obviously, the carcinogenesis version of “multiple roads lead to Rome” has been greatly amplified. Probably, those tax payers who are afflicted with cancer may not be satisfied by such an answer we provided that “I have found a way to make mice get cancer but I am not saying that any people got cancer in this way”. Actually, we cannot even explain why in most, if not all, of those animal models of solid cancers that have been created, only one or several overt tumors appear in the targeted organ, although all the cells in the organ have the same genotype. As mentioned earlier [54], the great discrepancy between tumor frequency with the number of animals as the denominator, which can be 100%, and that with the number of cells in the same organ as the denominator, which is much lower than 1 in a million, has hardly been addressed. What is used as the control for comparison is of importance. Numerous cells that surround, and share the same genotype with, the one or several tumors in the same organ of the same animal do not develop tumors; if they are used as controls, this probably rules out the genetically engineered genes as the drivers of the carcinogenesis.

## A cancer cell may fuse to another cell to gain survival advantage

Evolution requires an organisms’ reproduction, which proceeds in either a sexual or an asexual manner, each using a different genetic strategy to adapt to the environment and gain survival advantages. While almost all evolutionarily-higher animals reproduce sexually, lower-level organisms, typically bacteria, reproduce asexually. In general, the sexual manner helps the organisms to better adapt to the environmental change while still maintaining the integrity of the genome, thus the species, which is achieved by gaining beneficial mutations and purging away deleterious mutations. On the other hand, the asexual manner uses mutation loads to gain survival advantage and to adapt to the environmental change [86,87], but accumulation of mutations will easily lead to breaking the genome’s integrity, in contrast to the consequence of sexual propagation. Obviously, the above-described survival mechanism by accumulated mutations and ensuing Darwinian selections of those advantageous ones in cancer cells resembles an asexual evolution, which is evolutionarily lower and increases the cellular heterogeneity and thus increases the resource for selection of more-malignant phenotypes (Figure 2). However, accumulation of too much DNA damage may lead to the loss of too many genes needed for sustaining cell life and thus be lethal to some cancer cells (Figure 2). Actually, causing DNA damage, dubbed “genotoxicity”, is a central mechanism for many chemo drugs to elicit cancer-cell-specific killing [88], because normal cells are protected by a stronger DNA damage response and intact DNA repair mechanisms [89;90]. To survive, some cancer cells collaborate with each other [91-93] or with noncancerous stromal cells, such as to induce angiogenesis, usually via complicated cell-cell communications used for lower-level organisms like bacteria [94]. One extreme of such collaborations is cell fusion, i.e. a cancer cell fuses with another cancer cell, a normal stromal cell, or a macrophage to quickly obtain, in packages of chromosomes, life-sustaining genomic material [95-101]. Fusion to form a hybrid cell is a common manifestation of organismal evolution leading to new species of organisms, including some plants and animals. Today’s cotton as well as some strains of flies and fish are derived from this mechanism, i.e. fusion of species A and B to form species C with a sum of parental chromosomes [102-104]. Thus, cancer cell fusion can be regarded as another manifestation of this way of organismal evolution, which changes cancer cell’s genome at a much larger scale than mutation. Also importantly, this cell fusion somewhat resembles fertilization of an egg by a sperm and is thus in a sexual manner [55], i.e. an evolutionarily-higher-level mechanism for survival that cancer cells still retain [105] (Figure 2).

Normal hybrid somatic cells such as osteoclasts in the bone and syncytiotrophoblasts in the placenta that are formed by cell fusion usually do not replicate and remain polyploid. Therefore, in cancer how chromosomes in a hybrid cell are replicated and then distributed to the daughter cells remains an interesting question that is much unaddressed so far, although such cell fusion is known to be a cause of aneuploidy, a hallmark of cancer cells [106-108]. Moreover, since cells of many plants are polyploid whereas somatic cells of almost all animals are diploid [104], why cancer cells often resemble plant cells in being polyploid is also a conundrum to us. Nevertheless, yeasts, some evolutionarily-lower animals such as rotifers, and many species of plants reproduce sexually only during times of various forms of stress, including infections, and reproduce asexually in normal situations [109-114]. Therefore, this sexual manner of cell fusion may also occur more often or more easily when cancer cells are in a more stressful situation, such as during therapies. In fact, resistance to chemotherapy has been interpreted more in terms of gross changes in chromosome number (aneuploidy) or chromosome aberrations (instability) rather than of point mutations [115,116].

Fusion with a stromal cell and especially with a macrophage, both of which have strong intrinsic abilities to migrate, renders the hybrid cell competent to evade immune surveillance, to resist therapies, and, as well, to metastasize to and then colonize in distant body sites [106-108,117-119]. Moreover, in xenograft models where an animal was inoculated with human cancer cells, fusion of a human cell with a stromal cell of animal origin occurs often [97,98,120]. Therefore, how much the xenograft tumor reflects an in vivo situation of the parental human cancer cell line is partly dependent on how many animal-human hybrid cells have been formed.

## CONCLUSION

Sporadic carcinogenesis starts from reprogramming the death program of either a differentiated somatic cell or a tissue- or organ-specific stem cell, making the cell immortalized and autonomous. The immortalized cell actually incepts a new or quasi-new organism that lives like a parasite in the host patient and often undergoes a progressive simplification, constantly resulting in evolutionarily simpler cells with less differentiation than the normal cells of the parental tissue or organ. Like organismal evolution in Mother Nature, this cellular simplification that resembles a reverse evolution also occurs via Darwinian selections of those mutations with growth- or survival-advantages, from numerous ones that occur randomly and stochastically in immortalized cell and its derived cancer cells. Functional gain of growthor survival-sustaining oncogenes and functional loss of differentiation-sustaining tumor suppressor genes together constitute a best-known molecular profile of cancer cells and contribute to more-malignant phenotypes. However, these alterations are not drivers of carcinogenesis but, instead, are results from natural selection of advantageous mutations occurring as happenstances. This mutationload dependent mechanism for survival resembles an asexual evolution that increases cellular heterogeneity of evolutionarily lower organisms. In addition to this mechanism, cancer cells sometimes also choose to fuse with another cell to obtain beneficial genetic material. The cell fusion resembles fertilization of an egg by a sperm and thus resembles a sexual manner of evolution that is usually used by evolutionarily higher animals to gain beneficial mutations and purge away deleterious mutations for a purpose of better adapting environmental change while still maintaining the integrity of the species. Assigning oncogenes or tumor suppressor genes or their mutants as drivers, by engineering them under a DNA promoter we elected, to induce cancer in animals may somewhat coerce them to create numerous man-made oncogenic pathways that may not really be a course of sporadic cancer formations in the human.
